# Bed-Sharing at 3 Months and Breast-Feeding at 1 Year in Southern Brazil

**DOI:** 10.1016/j.jpeds.2009.04.037

**Published:** 2009-10

**Authors:** Iná S. Santos, Denise M. Mota, Alicia Matijasevich, Aluísio J.D. Barros, Fernando C.F. Barros

**Affiliations:** a Postgraduate Program in Epidemiology, Federal University of Pelotas, Pelotas, RS, Brazil; b Department of Maternal-Child Health, Federal University of Pelotas, Pelotas, RS, Brazil

**Keywords:** BF, Breast-feeding, CI, Confidence interval, DLP, Date of last period, IEN, National Economic Indicator (Brazil), LBW, Low birth weight, PR, Prevalence ratio, SIDS, Sudden infant death syndrome

## Abstract

**Objective:**

To investigate the association between bedsharing at age 3 months and breastfeeding (BF) at age 12 months.

**Study design:**

Almost all children born in Pelotas, Brazil in 2004 (99.2%) were enrolled in a cohort study. At birth, age 3 months, and age 12 months, mothers were interviewed to gather information on sociodemographic, reproductive, BF, and bedsharing characteristics. Bedsharing was defined as habitual sharing of a bed between mother and child for the entire night or part of the night. The analysis was limited to children from single births who were breastfed at 3 months. Multivariate analyses were carried out using Poisson regression.

**Results:**

Of 4231 live births, 2889 were breastfed at age 3 months. The prevalence of BF at age 12 months was 59.2% in the children who bedshared at 3 months and 44% in those who did not (adjusted prevalence ratio [PR] for weaning= 0.75; 95% confidence interval [CI] = 0.69-0.81; *P* < .001). Among children who were exclusively breastfed at 3 months, 75.1% of those who also bedshared were still breastfed at age 12 months, versus 52.3% of those who did not bedshare (adjusted PR = 0.63; 95% CI = 0.53- 0.75; *P* < .001). The adjusted PR was 0.74 (95% CI = 0.60-0.90; *P* = .003) in children who were predominantly breastfed and 0.83 (95% CI = 0.76-0.90; *P* < .001) in those who were partially breastfed.

**Conclusions:**

Bedsharing at 3 months protected against weaning up to age 12 months.

See editorials, p 461 and 462, and related article, p 475

International guidelines on infant nutrition elaborated by the World Health Organization and United Nations Children's Fund recommend exclusive breastfeeding (BF) for the first 6 months of life (breast milk only, without any other liquids or solids), and continued BF until age 2 years or older, combined with the timely and appropriate introduction of complementary foods.^1,2^ Compliance with these recommendations, especially during the first year of life, was identified as a major strategy for promoting infant survival by the Bellagio Child Survival Study Group.^3,4^

Many of the factors that influence how mothers feed their children and the time of onset and duration of BF are well known, as are various strategies for BF promotion.^5^ Proximity between mother and child is known to promote BF. The kangaroo-mother method for low birth weight (LBW) newborns^6^ and “rooming in,” one of the 10 steps of the Baby-Friendly Hospital Initiative,^7^ are both based on the beneficial effects of proximity on BF and on the mother–child relationship. These care strategies also decrease hospital infections and other causes of neonatal morbidity and mortality.

The effects of mother–child proximity on BF after discharge from the hospital also are under investigation. Like rooming in, bedsharing (mother and child sleeping in the same bed at night) has been shown to be associated with BF, even after controlling for confounders.^8^ Compared with other factors known to promote BF, however, the number of studies exploring this association is still small. Moreover, in recent years bedsharing has been discouraged because of its association with increased risk of sudden infant death syndrome (SIDS).^9-14^

The aim of the present study was to investigate the association between bedsharing at age 3 months and BF at age 12 months in the children of the 2004 birth cohort in Pelotas, Brazil.

## Methods

The birth cohort was obtained as described in detail previously.^15^ In brief, 99.2% of all children born in 2004 and living in the urban area of Pelotas and in the contiguous Jardim América neighborhood were enrolled in a cohort study. A perinatal interview was carried out in the hospital at the time of birth; mothers were interviewed again during home visits 3 months and 12 months after birth.

The perinatal study includes data on family socioeconomic status, schooling, age, skin color, parity, and mode of delivery. Socioeconomic status was divided into reference quintiles of Brazil's National Economic Indicator (IEN) for Pelotas.^16^ Mother's schooling was computed considering years of formal education with passing grades. Mother's age was collected in complete years at the time of delivery. Skin color was self-reported by the mother. Parity was categorized as 1, 2, 3, or ≥ 4. Mode of delivery was classified as normal (vaginal) or caesarian (c-section).

The following information on the newborn was recorded: sex, weight, gestational age, and history of neonatal problems. All newborns were weighed using a digital scale with 100-g precision and examined by the study team using the Dubowitz method.^17^ An infant born before 37 weeks of pregnancy was considered preterm. Gestational age was calculated using the algorithm proposed by the National Center for Health Statistics,^18^ with age estimated based on date of last period (DLP) adopted whenever available and consistent with birth weight, length, and head circumference according to the normal curves for these variables for each week of gestational age.^19^ When gestational age based on DLP was unknown or inconsistent, a clinical estimate of maturity based on the Dubowitz method was used. Neonatal problems reported by the mother (admission into neonatal intensive or semi-intensive care units) were recorded. Children born weighing < 2500 g were classified as LBW.

In the 3-month follow-up, mothers answered questions regarding paid work outside home (categorized as present or absent), diet, BF, and place where the child used to sleep, investigating the presence of bedsharing with adults or children. Bedsharing was defined as habitual sharing of a bed with another person (adult or child) for part of or the entire night. For purposes of the present evaluation, bedsharing was defined as habitually sharing a bed with the child's mother. BF pattern^20^ at 3 months was defined as exclusive BF, predominant BF (breast milk, herbal tea, fruit juice, and/or water), or partial BF (breast milk, herbal tea, fruit juice, and/or water, other liquids, and/or semisolids). At the 12-month follow-up, BF was again evaluated by means of a personal interview with the mother.

The present study included only infants from single births who were breastfed at age 3 months. Statistical analysis was carried out using Stata version 9.0 (StataCorp, College Station, Texas). The χ^2^ test was used to compare the frequencies of BF at age 12 months according to dichotomous exposures, and the linear trend χ^2^ test was used for ordinal exposures. Crude and adjusted relative risks were estimated using Poisson regression with robust variance.^21^ Multivariable analyses followed a hierarchical model, based on a theoretical causality framework constructed by the authors. In this model, age, mother's schooling, socioeconomic level, mother's working status at 3 months, and mother's skin color occupied the first, most distal level of determination, followed in the second level by parity, mode of delivery, and, more proximally, variables pertaining to the child. All variables were included in multivariate analyses; at each level, only those associated with the outcome at *P* ≤ .20 were kept in the model.

The study design was approved by the Research Ethics Committee of the Federal University of Pelotas School of Medicine. After being informed of the details of the study, each mother signed a term of informed consent for participation.

## Results

The study cohort comprised 4231 children born alive in Pelotas in 2004 (99.2%; 0.8% losses and refusals). In the 3-month follow-up visit, the mothers of 3985 children (94.2% of the original sample; 181 losses and refusals and 65 deaths) were interviewed; of these children, 2866 were breastfed. In the 12-month follow-up visit, there were 5.7% losses and refusals (from the original sample) and a total of 82 deaths, leaving 3907 children. For the entire cohort, the prevalence of BF dropped from 71.9% at age 3 months to 37.7% (n = 1442) at age 12 months.

Table I summarizes the maternal and child characteristics of children breastfed at age 3 months and the percentage of children in this group who were breastfed at age 12 months. The prevalence of habitual bedsharing with the mother at age 3 months was 48.3%. Table I shows a crude association between BF at age 12 months and maternal socioeconomic level, schooling, parity, skin color, mode of delivery, and working outside the home. Among child variables, associations with BF at age 12 months were detected for sex and bedsharing at age 3 months. The association between maternal schooling and continuity of BF in the first year of life was an inverse one; the higher the level of schooling, the lower the proportion of mothers that continued BF at age 12 months. The same was true for family socioeconomic level. Parity showed a direct association with the outcome; 48.5% of primiparas who breastfed at 3 months also breastfed at 12 months, compared with 55% of mothers with 4 or more previous deliveries. Girls, infants delivered vaginally, children of mothers who did not work outside the home in the third month, and children of black mothers were more likely to breastfeed at 12 months than their respective counterparts. Maintenance of BF to age 12 months also was more frequent in children whose mothers reported bedsharing at age 3 months; at age 12 months, 59.2% of infants who bed-shared at 3 months were still breast-fed, compared with 44% of those who did not bed-share (*P* < .001).

Table II presents the crude and adjusted effects of bedsharing on weaning at age 12 months. The pattern of BF at age 3 months was available for 3836 children: 32.5% (1246) were exclusively breastfed, 17.8% (683) were predominantly breastfed, 22.5% (867) were partially breastfed, and 27.1% (1040) were no longer breastfed. The crude association between bedsharing at 3 months and BF at 12 months remained unaltered after controlling for confounders. The adjusted analysis included all maternal and child variables, and the final model included mother's age, socioeconomic classification, mother's skin color, mother working outside the home, and type of delivery, along with infant's sex and LBW. Bedsharing at 3 months was associated with an adjusted 25% reduction (95% confidence interval [CI] = 19%-31%) in the risk of weaning at age 12 months.

An analysis of the effect of bedsharing stratified by BF pattern showed that children who were exclusively breastfed had a reduced risk of weaning of 37% (95% CI = 25%-47%), after allowing for socioeconomic classification, mother's skin color, parity, type of delivery, and child's sex. In children who were predominantly breastfed, after controlling for socioeconomic classification and mother's schooling, this reduction was 26% (95% CI = 10%-40%), and in children who were partially breastfed, it was 17% (95% CI = 10%-24%), adjusted for mother's age, socioeconomic classification, and mother's skin color (Table II).

In children who were exclusively breastfed at 3 months and who also bedshared, 75.1% continued to be breastfed at 12 months, in contrast to 52.3% of those who did not bedshare (*P* < .001). In those who were predominantly breastfed at 3 months, the prevalence of BF at 12 months was 70.1% in the who bedshared and 48.3% in those who did not (*P* < .001). In children who were partially breastfed at 3 months, these proportions were 37.9% and 18.6%, respectively (*P* < .001).

The Figure shows the BF curve until age 12 months for children in the presence and absence of bedsharing at 3 months, after adjustment for the variables listed in Table II. As shown, the weaning rate until age 12 months was faster in children who did not bedshare at age 3 months.

## Discussion

After allowing for confounders, habitual bedsharing with the mother at age 3 months protected against weaning until age 12 months, independent of the BF pattern (exclusive, predominant, or partial) at age 3 months. The protective effect was stronger in those infants who were exclusively breastfed. However, maternal personality characteristics, together with parental lifestyle, may be associated with bedsharing and also contribute to increased duration of BF.^22^ These variables were not been addressed in the present analyses, representing a limitation of this study. In addition, this study was conducted in a specific area of a middle-income country, which might limit generalization of the findings.

The protective effect of bedsharing on the risk of weaning is in agreement with the findings reported from other studies. McKenna et al^23^ studied mother–child pairs with infants age 3 to 4 months who were exclusively breastfed during the night: 20 pairs who habitually bedshared and 15 pairs who did not. In those who bedshared, the total time spent in BF was 3-fold greater, the number of BF sessions was 2-fold greater, and individual BF sessions were 39% longer. Clements et al,^8^ in a sample of 700 infants, found an association between bedsharing and longer duration of BF even after controlling for confounders. Okami et al,^24^ in a sample of 205 European-American families followed since the third trimester of pregnancy, found an association between bedsharing at age 5 months and duration of BF. Vogel et al,^25^ in a cohort of 350 newborns followed up to 1 year, found an association between bedsharing at 3 months and a reduced risk for shorter duration of BF. Ball,^26^ in a sample of 253 families, found that bedsharing promoted BF in the first 4 months of life.

Physical proximity to and contact with the mother during sleep expedite the mother's response to feeding requests from the child, and also provides psychological comfort to the child and parents. Studies assessing the prevalence of bedsharing have identified it as a fairly common practice in different countries. A US study of more than 10000 infants age 1 month identified a bedsharing prevalence of 22%.^27^ Studies in New Zealand and Australia found bedsharing prevalences exceeding 40%.^28,29^ Our data show a high prevalence of bedsharing in our settings as well: 46.4% at 3 months and 43.7% at 12 months. At 12 months, > 60% of children who bedshared slept with both parents in the same bed.^30^

The benefits and harms to children associated with bedsharing have been the subject of several investigations, most of which were designed to identify potential risk factors for SIDS.^14^ The interaction between bedsharing and smoking for risk of SIDS also has been explored in several studies. Although bedsharing has been discouraged due to the risk of SIDS,^9-14^ some studies have shown an increased risk only in infants whose mothers smoke.^28,31,32^ Epidemiologic studies, including a meta-analysis, have found an association between bedsharing and lower risk of SIDS, especially in infants who were exclusively breastfed until age 4 months.^33,34^ To date, however, there is insufficient evidence to suggest that BF is a protective factor against SIDS.

Besides BF, studies on the child-related benefits of bedsharing have mainly explored sleep–wake patterns. Infants who bedshare have an increased number of awakenings during the night compared with those who do not bedshare, suggesting that an infant's hability to rouse may be protective against SIDS.^35-37^

Until a definitive position with respect to the risks and benefits of bedsharing is available, parents should be alerted as to unsafe practices during their child's sleep. These include cigarette smoke in the environment and maternal smoking;^28,32,38-40^ sharing of sofas or other types of bedding made of soft, depressible materials;^12,13^ and use of alcohol or other drugs that alter the consciousness of the adult sharing a bed with the child.^41^ In addition, parents should be informed about safe sleep practices, including placing the child in a supine position^42^ and on a firm surface, avoiding sofas, pillows, cushions, and lose blankets.^9,12,13,42^ The advantages and disadvantages of bedsharing in different settings need to be explored, to provide sound scientific evidence supporting the promotion or discouragement of this practice.

## Figures and Tables

**Figure:**
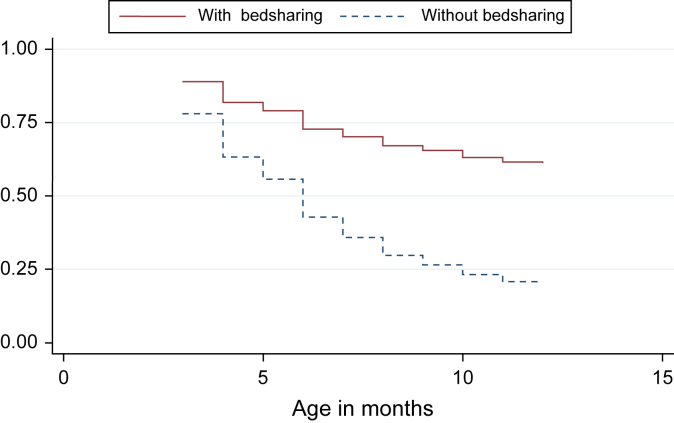
BF at age 12 months according to bedsharing status at age 3 months, adjusted for schooling, parity, maternal work, mother's skin color, mode of delivery, and socioeconomic level in the 2004 Pelotas birth cohort.

**Table I:** Sample description and frequency of BF at age 12 months, according to maternal and neonatal child characteristics, among children breastfed at 3 months, 2004 Pelotas birth cohort (n = 2866)

Variable	Total n (%)	BF at age 12 months, n (%)
Mother's age, years (n = 2761)		*P* = .14
< 20	461 (16.7)	243 (52.7)
20-29	1388 (50.3)	692 (49.9)
30-39	823 (29.8)	426 (51.8)
≥ 40	89 (3.2)	55 (61.8)
Mother's schooling, years (n = 2730)		*P* = .002∗
0-4	393 (14.4)	219 (52.7)
5-8	1058 (38.8)	560 (52.9)
9-11	983 (36.0)	488 (49.6)
≥ 12	296 (10.8)	134 (45.3)
Parity (n = 2761)		*P* = .03∗
1	1055 (38.2)	512 (48.5)
2	761 (27.6)	405 (53.2)
3	450 (16.3)	228 (50.7)
≥ 4	495 (17.9)	272 (55.0)
Mode of delivery (n = 2762)		*P* = .01
Vaginal	1520 (55.0)	812 (53.4)
C-section	1242 (45.0)	605 (48.7)
Socioeconomic classification (IEN), quintiles of reference for Pelotas (n = 2760)		*P* < .001∗
1 (20% poorest)	619 (22.4)	350 (53.5)
2	555 (20.1)	285 (51.4)
3	608 (22.0)	316 (52.0)
4	517 (18.7)	263 (50.9)
5 (20% richest)	461 (16.7)	202 (43.8)
Mother working outside the home at 3 months (n = 2762)		*P* = .002
Yes	309 (11.2)	133 (43.0)
No	2453 (88.8)	1284 (52.3)
Mother's skin color (self-referred) (n = 2731)		*P* < .001
White	1685 (61.7)	815 (48.4)
Black	492 (18.0)	283 (57.5)
Mixed	554 (20.3)	302 (54.5)
Child's sex (n = 2762)		*P* = .03
Male	1421 (51.5)	700 (49.3)
Female	1341 (48.5)	717 (53.5)
Preterm (n = 2761)		*P* = .77
Yes	340 (12.3)	172 (50.6)
No	2421(87.7)	1245 (51.4)
Low birthweight (< 2500 g) (n = 2762)		*P* = .29
Yes	191 (6.9)	91 (47.6)
No	2571 (93.1)	1326 (51.6)
Neonatal problems (n = 2756)		*P* = .55
Yes	278 (10.1)	138 (49.6)
No	2478 (89.9)	1277(51.5)
Bedsharing at 3 months (n = 2636)		*P* < .001
Yes	1383 (48.3)	787 (59.2)
No	1483 (51.7)	630 (44.0)

∗ Linear trend test.

**Table II:** Crude and adjusted effect of bedsharing at age 3 months (prevalence ratios) in preventing weaning at age 12 months, 2004 Pelotas birth cohort

	PR	95% CI	P
All children (n = 2762)			
Crude	0.73	0.67-0.79	< .001
Adjusted∗	0.75	0.69-0.81	< .001
Children with exclusive BF (n = 1246)			
Crude	0.60	0.51-0.71	< .001
Adjusted†	0.63	0.53-0.75	< .001
Children with predominant BF (n = 683)			
Crude	0.68	0.56-0.83	< .001
Adjusted‡	0.74	0.60-0.90	.003
Children with partial BF (n = 867)			
Crude	0.80	0.73-0.87	< .001
Adjusted§	0.83	0.76-0.90	< .001

∗ Adjusted for mother's age, socioeconomic level, maternal working outside the home at 3 months, mother's skin color, mode of delivery, child's sex, and low birth weight.

† Adjusted for socioeconomic level, mother's skin color, parity, mode of delivery, and child's sex.

‡ Adjusted for socioeconomic level and mother's schooling.

§ Adjusted for mother's age, socioeconomic level, and mother's skin color.
